# Generating Low-Earth Orbit Satellite Attitude Maneuver Profiles Using Deep Neural Networks

**DOI:** 10.3390/s23104650

**Published:** 2023-05-11

**Authors:** Seok-Teak Yun

**Affiliations:** Division of KOMPSAT-7 Program, Korea Aerospace Research Institute, Daejeon 34133, Republic of Korea; yungun@kari.re.kr; Tel.: +82-42-860-2974

**Keywords:** LEO satellites, reference attitude profiles, target-pointing attitude, data-based learning, deep neural networks

## Abstract

To perform Earth observations, low-Earth orbit (LEO) satellites require attitude maneuvers, which can be classified into two types: maintenance of a target-pointing attitude and maneuvering between target-pointing attitudes. The former depends on the observation target, while the latter has nonlinear characteristics and must consider various conditions. Therefore, generating an optimal reference attitude profile is difficult. Mission performance and satellite antenna position-to-ground communication are also determined by the maneuver profile between the target-pointing attitudes. Generating a reference maneuver profile with small errors before target pointing can enhance the quality of the observation images and increase the maximum possible number of missions and accuracy of ground contact. Therefore, herein we proposed a technique for optimizing the maneuver profile between target-pointing attitudes based on data-based learning. We used a deep neural network based on bidirectional long short-term memory to model the quaternion profiles of LEO satellites. This model was used to predict the maneuvers between target-pointing attitudes. After predicting the attitude profile, it was differentiated to obtain the time and angular acceleration profiles. The optimal maneuver reference profile was obtained by Bayesian-based optimization. To verify the performance of the proposed technique, the results of maneuvers in the 2–68° range were analyzed.

## 1. Introduction

In recent decades, low-Earth orbit (LEO) satellites have been developed and launched for various purposes. LEO satellites use attitude maneuvers to perform Earth observations [1,2]. Attitude maneuvers can be classified into two types: performance during the mission (maintenance of a target-pointing attitude) and maneuvering between missions (maneuvering between target-pointing attitudes). The pointing stability of the line of sight for Earth observations is the critical mission performance factor, and the attitude control performance of the satellite, pointing knowledge, and rate drift are also important factors [3,4]. The attitude profile of the target-pointing segment of the mission is determined by the user’s observation requirements. Typically, the target-pointing attitude is designed to have low angular rates to minimize drift during observations [2,5]. Observation mission requirements, such as the imaging area and location, are used as input conditions to generate the attitude profile of the target-pointing segment. The attitude profile of the target-pointing duration generated in this way has a deterministic solution depending on the observation target. Therefore, deviations in the performance of the attitude profile of the target-pointing segment of the mission are generally insignificant.

In contrast, the attitude maneuver profile between target pointings has various initial and final attitude conditions and must be executed within certain constraints, such as the allowable range of angular rates and maneuver time requirements [6,7]. Therefore, depending on the cost function and generation logic, significant variations in the maneuver time and attitude error at the starting point of the target pointings can occur. Generally, the maneuvering between target pointings is a critical indicator of a satellite’s mission performance capability and requires optimization [7,8]. However, without an initial guess, the optimization process can be time-consuming.

Because the demand for high-agility satellites that maximize the observation time is increasing, many satellites that minimize the maneuver time between the target pointings and attitude error at the initiation of the mission are being developed [9,10,11]. Additionally, as demand for satellite imagery for urgent missions (e.g., disaster response) increases, the need to maximize mission performance through rapid maneuvers also increases [12]. Therefore, a satellite’s agile performance is crucial to all commercial, military, and government-led satellite developments because it indicates the satellite’s ability to quickly change its attitude to meet specific mission requirements [12,13]. 

To achieve a high level of satellite agility, it is necessary to consider factors such as actuators capable of producing high torque, which minimize the moment of inertia (MOI) of the satellite, and the sending of reference attitude commands for maneuvering between targets [6,7]. Actuators, such as high-torque reaction wheels and control moment gyros, are the hardware that needs to be considered in the early stages of satellite development. They exhibit characteristics with limiting factors, such as reliability, cost, and schedule. The weight and size of the payload tend to increase as the observation performance increases, which limits the potential for the reduction of the MOI through optimization. In addition, a control system that combines feedforward and feedback control methods (rather than a single feedback controller) needs to be designed to achieve the shortest maneuvering time [14,15]. In this control system, the command for the satellite’s attitude maneuver is provided as feedforward, whereas any disturbance to the maneuvering is controlled as feedback [7]. Finally, to minimize disturbances, the reference attitude command for the maneuver between target pointings should be developed to minimize the attitude error (quaternion and angular rate) at the start of the mission under the constraint of the satellite’s maneuvering limits [12]. 

The attitude maneuver profile of an LEO satellite is used as the input for driving the antenna to transmit mission data, and a reference profile for the antenna maneuver is created [16,17,18]. Observation missions generate a large number of data owing to high-resolution images and also require a high data transfer rate [16]. As a result, limited error ranges in the beam width are required to accommodate the high data transfer rates. To generate an accurate ground station pointing profile, it is necessary to have the attitude information of the satellite while the ground station is in contact with it, including the target-pointing maneuvers. Owing to the difficulty in generating the attitude reference profile for the interval between target pointings, the attitude information is often not generated or provided by the ground station, and the range of allowable errors in the beam width is limited, owing to the need for high data transfer rates. Therefore, if the attitude reference profile for the duration of the target pointing is generated only on the ground, there may be an antenna pointing error between the ground prediction and actual attitude during the maneuver, which leads to problems in the continuous transmission of the data [18]. In particular, steady and stable attitude profile generation is essential for modern high-resolution satellites that transmit large datasets. If the generated profile does not match the initial attitude and angular rate at the beginning of the mission, an additional stabilization time may be required for initializing the target pointing, limiting the maximum number of possible missions [2]. Therefore, generating the optimal reference profile for maneuvering between the target pointing of the observation satellite is essential for maximizing the performance of the satellite mission and driving the antenna for transmitting the data. However, it is difficult to find a generalized solution owing to satellite limiting conditions, nonlinearity, and various startup and end conditions [4,7,8,10,19].

In this study, we optimize the reference attitude command for the pointing maneuver between target pointings via a control method with feedforward and feedback control, and we propose a technique for developing a reference attitude command that optimizes the maneuvering between target-pointing intervals using data training to predict the attitude during maneuvering.

A summary of the contributions of this study is as follows:

- This paper proposes a ground-based technique for generating optimal reference profiles for maneuvers between target pointings of LEO observation satellites.
- The proposed technique predicts the quaternions of the maneuver through data-based learning and uses the predicted value as an initial guess for optimization to generate the maneuver profile.
- To demonstrate the performance of the proposed technique, the error of the starting point of the target pointing according to various maneuver angles, start and end angular rates are analyzed, and its performance is compared with the existing technique.

The remainder of this paper is organized as follows. Section 2 describes the background and constraints of the maneuvering of LEO satellites. Section 3 explains the approach for generating reference attitude profiles for maneuvering between target pointings using the proposed data-based initial guess estimation and optimization technique. Section 4 describes the network and training results of the data-based initial guess estimation. Section 5 presents the attitude profile generation results based on the angle and angular rate. Finally, the conclusions are summarized in Section 6. 

## 2. Background 

### 2.1. LEO Satellite Attitude Guidance Profile 

**Figure 1:**
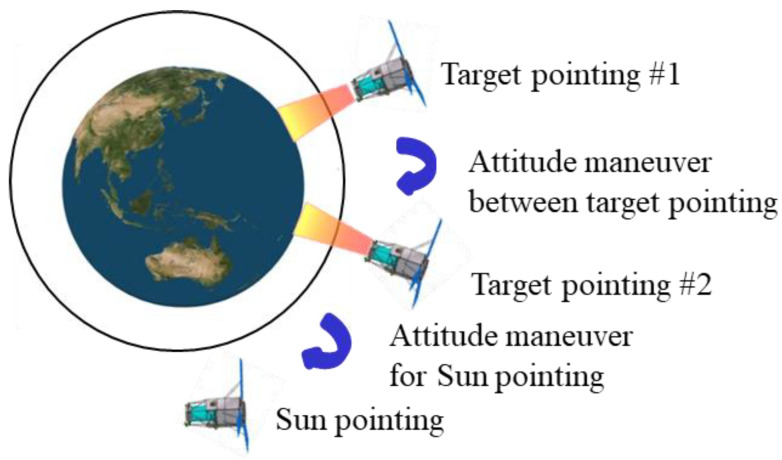
Attitude mode of low-Earth orbit (LEO) satellite.

Research on satellite maneuver optimization has been conducted since the 1970s [3,4,5]. The satellite’s actuator performance limitations, transmission capacity in orbit, and throughput should be considered for the optimal maneuvering of LEO satellites. A depiction of attitude maneuvers required for observation missions of LEO satellites is illustrated in Figure 1. Generally, attitude maneuvering for an LEO satellite can be divided into the target pointing for the duration of the observation mission, maneuvering between target pointings, and sun pointing for maximum power generation. The target-pointing duration is the period in which the attitude is determined according to the user’s desired observation target (e.g., Earth, star, or other region in space). Therefore, this duration generally requires minimal or no attitude changes. Furthermore, the attitude required for the target-pointing duration is determined by the satellite’s position, velocity, observation target, and observation interval.

The duration of attitude maneuvers between two target points varies according to the maneuvering constraints and attitude generation method. It is difficult to determine this duration because of the differences in boundary conditions between the attitudes and angular velocities at the end of the previous target pointing and the beginning of the next pointing [19,20,21]. Therefore, because of the different initial and final maneuvering conditions, the available torque and angular rate of the satellite are limited, and it is difficult to obtain a generalized solution [10]. Generally, as the time for attitude maneuvering between target pointings decreases, the number of possible missions increases. In this study, we optimized the attitude maneuvering between target pointings using data-based learning to maximize LEO mission performance.

Finally, after a LEO satellite completes its observations, a sun-pointing attitude maneuver is required for maximizing the power generation [22]. 

### 2.2. LEO Satellite Attitude Guidance Profile Constraints

The maximum allowable values for the angular acceleration and angular rate should be used to maximize the attitude maneuver performance of an LEO satellite. The maximum torque and momentum of the actuators primarily limit the maximum angular acceleration and angular rate of an LEO satellite [7,10,19]. There may be additional constraints on the angular rate to ensure the performance of the GPS receivers and star trackers.

**Figure 2:**
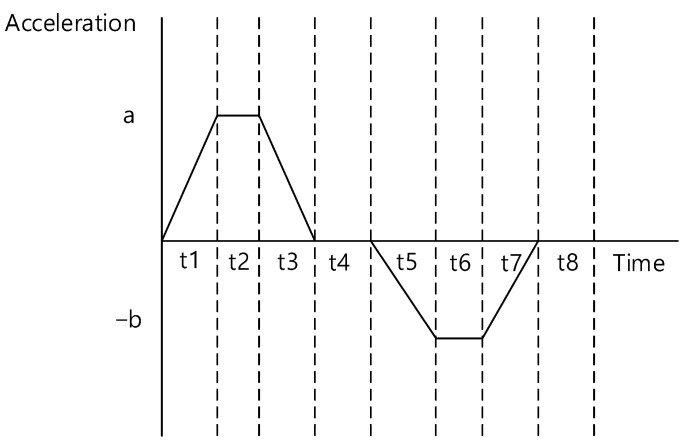
Attitude upload profile between target pointings of LEO satellites.

When generating the attitude profiles for target pointings and maneuvering between target pointings, there may be an increase in the throughput of the flight software, which can lead to difficulties in performing complex missions. Furthermore, this approach may not be applicable to satellites in orbit. Therefore, we considered a ground-based technique for generating a reference attitude maneuver profile for operational applications. The reference attitude maneuver profile, which is generated on the ground and uploaded to the satellite, should minimize the increase in throughput when restored onboard. In particular, the contact time with LEO satellites is limited [23]. As a result, the upload time for mission execution, scheduled command sets for the next satellite contact, and playback execution for receiving satellite data during the time in which the satellite is inaccessible are also limited. Therefore, it is important to also minimize the data size uploaded. Accordingly, we developed an optimization approach to provide LEO satellites with reference data for attitude maneuvers between target pointings using the acceleration profiles for each axis, as shown in Figure 2. In other words, the size of the data sent to the satellite was reduced by using the time information ($t_{1}\;to$ $t_{8}$), and the maximum and minimum values of the acceleration profile for each axis (a and b) are shown in Figure 2. Then, onboard the satellite, the acceleration was integrated to create a reference profile for the satellite’s angular rate and attitude to maneuver the satellite. 

## 3. Proposed Technique for Generating Attitude Reference Profiles

Section 2 described the difficulty in obtaining a maneuver profile in a short time, considering all the limiting factors. This occurs because the satellite’s attitude kinematics model is nonlinear, and previous research-derived solutions used optimal control theory [24,25,26,27]. However, in this approach, the initial guesses influence the quality of the potential solutions. Therefore, we proposed a technique that uses a data-driven deep neural network (DNN) to obtain the initial guesses and then is optimized to minimize the pointing error and maneuvering time as the satellite points to its new target.

First, the upload profile of the LEO satellite was created using angular acceleration and time data, as mentioned in Section 2. The values of the angular acceleration generated for each time, shown in Figure 2, are defined as

(1) $$a\left(t\right)=a_{0}+J\cdot t,\;0<t<t_{1},$$

(2) $$a\left(t\right)=a_{0}+J\cdot t_{1}=a_{12},\;t_{1}<t<t_{2},$$

(3) $$a\left(t\right)=a_{12}-J\cdot \left({t-t}_{2}\right),\;t_{2}<t<t_{3},$$

(4) $$a\left(t\right)=a_{12}-J\cdot \left({t_{3}-t}_{2}\right)=a_{34},\;t_{3}<t<t_{4},$$

(5) $$a\left(t\right)=a_{34}-J\cdot \left({t-t}_{4}\right),\;t_{4}<t<t_{5},$$

(6) $$a\left(t\right)=a_{34}-J\cdot \left({t_{5}-t}_{4}\right)=a_{56},\;t_{5}<t<t_{6},$$

(7) $$a\left(t\right)=a_{56}+J\cdot \left({t-t}_{6}\right),\;t_{6}<t<t_{7},$$

where $a_{0}$ is the initial acceleration value and J is the maximum jerk value of satellite.

The angular velocities for each segment from *t*_1_ to *t*_7_ are given as

(8) $$\omega \left(t\right)=a_{0}\cdot t+\frac{1}{2}J\cdot t^{2},\;0<t<t_{1},$$

(9) $$\omega_{1}=a_{0}\cdot t_{1}+\frac{1}{2}J\cdot {t_{1}}^{2},$$

(10) $$\omega \left(t\right)=\omega_{1}+a_{12}\cdot \left({t-t}_{1}\right),\;t_{1}<t<t_{2},$$

(11) $$\omega_{2}=\omega_{1}+a_{12}\cdot \left({t_{2}-t}_{1}\right),$$

(12) $$\omega \left(t\right)=\omega_{2}+a_{12}\cdot \left({t-t}_{2}\right)-\frac{1}{2}J\cdot {\left({t-t}_{2}\right)}^{2},\;t_{2}<t<t_{3},$$

(13) $$\omega_{3}=\omega_{2}+a_{12}\cdot \left({t_{3}-t}_{2}\right)-\frac{1}{2}J\cdot {\left({t_{3}-t}_{2}\right)}^{2},$$

(14) $$\omega \left(t\right)=\omega_{3}+a_{12}\cdot \left({t-t}_{3}\right),\;t_{3}<t<t_{4},$$

(15) $$\omega_{4}=\omega_{3}+a_{34}\cdot \left({t_{4}-t}_{3}\right),$$

(16) $$\omega \left(t\right)=\omega_{4}+a_{34}\cdot \left({t-t}_{4}\right)-\frac{1}{2}J\cdot {\left({t-t}_{4}\right)}^{2},\;t_{4}<t<t_{5},$$

(17) $$\omega \left(t\right)=\omega_{4}+a_{34}\cdot \left({t_{5}-t}_{4}\right)-\frac{1}{2}J\cdot {\left({t_{5}-t}_{4}\right)}^{2},$$

(18) $$\omega \left(t\right)=\omega_{5}+a_{56}\cdot \left({t-t}_{5}\right),\;t_{5}<t<t_{6},$$

(19) $$\omega_{6}=\omega_{5}+a_{56}\cdot \left({t_{6}-t}_{5}\right),$$

(20) $$\omega \left(t\right)=\omega_{6}+a_{56}\cdot \left({t-t}_{6}\right)+\frac{1}{2}J\cdot {\left({t-t}_{6}\right)}^{2},\;t_{6}<t<t_{7},$$

Finally, the angles for duration are given by

(21) $$\theta \left(t\right)=\frac{1}{2}a_{0}\cdot t+\frac{1}{6}J\cdot t^{3},\;0<t<t_{1},$$

(22) $$\theta_{1}=\frac{1}{2}a_{0}\cdot t_{1}+\frac{1}{6}J\cdot t_{1},$$

(23) $$\theta \left(t\right)=\theta_{1}+\omega_{1}\left({t-t}_{1}\right)+\frac{1}{2}a_{12}\cdot {\left({t-t}_{1}\right)}^{2},\;t_{1}<t<t_{2},$$

(24) $$\theta_{2}=\theta_{1}+\omega_{1}\left({t_{2}-t}_{1}\right)+\frac{1}{2}a_{12}\cdot {\left({t_{2}-t}_{1}\right)}^{2},$$

(25) $$\theta \left(t\right)=\theta_{2}+\omega_{2}\left({t-t}_{2}\right)+\frac{1}{2}a_{12}\cdot {\left({t-t}_{2}\right)}^{2}-\frac{1}{6}J\cdot {\left({t-t}_{2}\right)}^{3},\;t_{2}<t<t_{3},$$

(26) $$\theta_{3}=\theta_{2}+\omega_{2}\left({t_{3}-t}_{2}\right)+\frac{1}{2}a_{12}\cdot {\left({t_{3}-t}_{2}\right)}^{2}-\frac{1}{6}J\cdot {\left({t_{3}-t}_{2}\right)}^{3},$$

(27) $$\theta \left(t\right)=\theta_{3}+\omega_{3}\left({t-t}_{3}\right)+\frac{1}{2}a_{34}\cdot {\left({t-t}_{3}\right)}^{2},\;t_{3}<t<t_{4},$$

(28) $$\theta_{4}=\theta_{3}+\omega_{3}\left({t_{4}-t}_{3}\right)+\frac{1}{2}a_{34}\cdot {\left({t_{4}-t}_{3}\right)}^{2},$$

(29) $$\theta \left(t\right)=\theta_{4}+\omega_{4}\left({t-t}_{4}\right)+\frac{1}{2}a_{34}\cdot {\left({t-t}_{4}\right)}^{2}-\frac{1}{6}J\cdot {\left({t-t}_{4}\right)}^{3},\;t_{4}<t<t_{5},$$

(30) $$\theta_{5}=\theta_{4}+\omega_{4}\left({t_{5}-t}_{4}\right)+\frac{1}{2}a_{34}\cdot {\left({t_{5}-t}_{4}\right)}^{2}-\frac{1}{6}J\cdot {\left({t_{5}-t}_{4}\right)}^{3},$$

(31) $$\theta \left(t\right)=\theta_{5}+\omega_{5}\left({t-t}_{5}\right)+\frac{1}{2}a_{56}\cdot {\left({t-t}_{5}\right)}^{2},\;t_{5}<t<t_{6},$$

(32) $$\theta_{6}=\theta_{5}+\omega_{5}\left({t_{6}-t}_{5}\right)+\frac{1}{2}a_{56}\cdot {\left({t_{6}-t}_{5}\right)}^{2},$$

(33) $$\theta \left(t\right)=\theta_{6}+\omega_{6}\left({t-t}_{6}\right)+\frac{1}{2}a_{56}\cdot {\left({t-t}_{6}\right)}^{2}+\frac{1}{6}J\cdot {\left({t-t}_{6}\right)}^{3},\;t_{6}<t<t_{7},$$

The attitude and angular rate profile of the satellite up to *t*_7_ were generated onboard the satellite using the uploaded acceleration and time data. The commands for satellite attitude maneuvering were provided in quaternion form instead of Euler angles to avoid gimbal locks and other issues. The attitude profile for the maneuvering segment was converted into a quaternion form for training using a direction cosine matrix (DCM).

The format of the data uploaded to the LEO satellites and used for maneuvering between target pointings is shown in Table 1. Table 1 shows that the maximum and minimum acceleration values (*a*, *b*) and time duration data ($t_{1}$ to $t_{7}$) are used for each axis. The value of *t*_8_ was determined at the start of the mission according to the mission design requirements for the satellite, and it was defined as the difference between the user-controlled maneuver time and the time interval from $t_{1}$ to $t_{7}$. To account for the size of the uploaded data, the same time ($t_{1}$ to $t_{8}$) values were used for the roll, pitch, and yaw axes. In other words, all three axes had the same timeline, and only the acceleration values were generated differently.

The proposed technique that employs DNN-based prediction uses data-based learning to obtain the initial guess for optimization. To perform data-based learning, it was necessary to preprocess the data, separate the data for training and validation, design the network and optimize it for learning, and validate the predictions.

In the preprocessing stage, the input and output features must be selected. For this process, it was necessary to select features that correlated with the output feature. As mentioned in Section 2, to reduce the dimension of the features and improve the accuracy of the model, the next point quaternion (${Q1}_{next\;point}$, ${Q2}_{next\;point},{Q3}_{next\;point},{Q4}_{next\;point}$) was selected as the output feature. For the input feature, the Earth-centered, Earth-fixed (ECEF) frame’s position and velocity, the current point quaternion (${Q1}_{current\;point},{Q2}_{current\;point},{Q3}_{current\;point},{Q4}_{current\;point}$) of the satellite’s orbital parameters, and the relative angle and rate difference between the target pointing’s initial position for the next mission and the current time were selected. The selected features are presented in Table 2. 

Because the values of the selected features have different ranges, the values were normalized to optimize learning via the gradient descent. Preprocessing was executed using the min—max method to process each feature uniformly according to

(34) $$\frac{X_{traning\;data}-\min(X_{traning\;data})}{\max\left(X_{traning\;data}\right)-\min(X_{traning\;data})},$$

where $\min(X_{traning\;data})$ and $\max\left(X_{traning\;data}\right)$ are the minimum and maximum values of each training data, respectively.

To proceed with the training, the dataset was generated and divided into four parts to train the modeling network, select the parameters and network, test the quaternion prediction performance, and verify the maneuver profiles. The dataset was generated using a satellite dynamic attitude simulator under the following conditions: the maximum jerk was 9 ${deg/s}^{3}$, the maximum angular acceleration was 0.9 ${deg/s}^{2}$, the maximum angular rate was 2.9 deg/s, and the MOI values of the satellite for each axis were assumed to be 2500 ${kg\;m}^{2}$, 2500 ${kg\;m}^{2}$, and 1000 ${kg\;m}^{2}$. The optimal maneuver quaternion time-series dataset was obtained by performing trial-and-error with dynamic simulations to achieve the minimum feasible maneuver time and error. 

The dataset was composed of 1000 time-series samples with different initial and final orientations and angular velocities, of which 60, 10, 10, and 20% were used for training, modeling the network, and selecting the parameters, testing the quaternion prediction performance based on the trained model, and verifying the performance of the generated profiles, respectively. 

**Figure 3:**
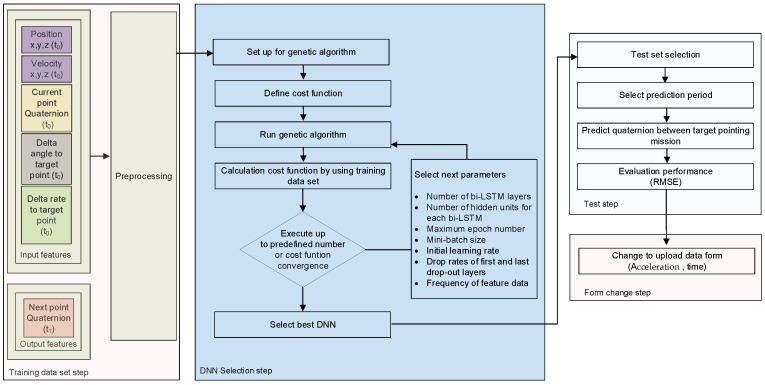
Training process for quaternion prediction of maneuvering duration.

The procedure for creating a quaternion prediction model for maneuvering is shown in Figure 3. The optimal network and parameters were selected using a genetic algorithm to minimize the error of the time-series prediction results during the maneuver period based on the training and parameter selection datasets. The cost function for selecting the optimal network and parameters for training was designed to minimize the error of the time-series prediction results during the maneuver period, as indicated by

(35) $$cost\;funtion\;for\;traning=\sqrt{\frac{1}{n}\cdot \sum {\left(Q_{verification}-Q_{Prediction}\right)}^{2}},$$

where $Q_{verification}$ is the reference quaternions of the verification dataset, n is the number of the verification quaternions data, and $Q_{Prediction}$ is the predicted quaternion values of the DNN model.

The selection factors for the network and parameters used to model the quaternion include the number of bidirectional long short-term memory (bi-LSTM) layers, number of hidden units for each bi-LSTM, maximum epoch number, mini-batch size, initial learning rate, drop rates of the first and last drop-out layers, and frequency of the feature data [28,29,30]. As the reverse order of time-series data is also meaningful, learning using bi-LSTM was used because of its high prediction accuracy of time-series data [31,32]. Next, the frequency of the feature data was chosen as the selection factor for optimization. If the frequency of each feature data is too dense, training can become time-consuming, and overfitting may occur. Conversely, if the frequency is extremely low, the accuracy of the training model may decrease.

**Figure 4:**
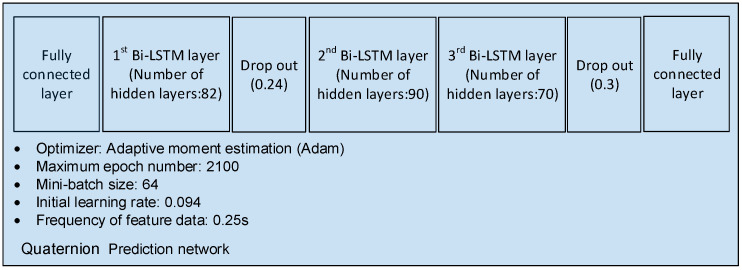
Maneuver reference profile generation network for LEO satellites.

The factors for developing the network were selected using a genetic algorithm (GA) to modify each parameter and find the DNN that minimized the cost function within a specified number of iterations (in this case, 500). The DNN architecture determined using the GA is shown in Figure 4.

The process proposed for generating the maneuver profile of an LEO satellite between its target pointings was performed in two stages. In the first stage, an initial guess for the profile was obtained for optimization. The satellite’s position, velocity, relative angle, angular rate difference to the target point, and quaternion at the start of the maneuver were fed to the pretrained DNN to output the next point’s quaternion. Then, the next point’s position, velocity, relative angle, angular rate difference to the target point, and the generated quaternion were sent as current information to predict the quaternion of the next point. This process was repeated to predict the quaternion until the next target pointing’s starting point was reached. The aim of this process was to reproduce the quaternion from the initial point to the final point of the maneuver. The predicted quaternion values were then converted into angular accelerations and time profiles for each axis, which were uploaded to the LEO satellite via Euler angle transformation and differentiation.

In the second stage, the angular acceleration and time profiles of each axis obtained as initial guesses in the first stage were used for optimization. The cost function for this optimization process utilized the quaternion at the end of the generated profile, maneuver time, and angular rate error. The comparison point of the cost function affected the final performance. If the calculation point of the cost function is set as the initial target pointing of the mission, errors may occur at the beginning of the mission, requiring additional time for stabilization depending on the attitude control. Therefore, $t_{7}$ in Figure 2 was selected as the calculation point for the cost function to secure additional stabilization time for optimization.

As shown in Figure 2, the generated profile only has angular acceleration values up to $t_{7}$. Therefore, to calculate the quaternion and angular rate error at the end of the profile for optimization, the quaternion and rate values at $t_{7}$ generated up to that point was compared using the quaternion and rate information at the start of the mission, and it was backpropagated for a duration of *t*_8_ as follows:

(36) $${\theta \left(t\right)}_{back}=\omega_{mission}\left({t-t}_{8}\right),$$

where ${\theta \left(t\right)}_{back}$ is the backpropagated angle from the start of target pointing and $\omega_{mission}$ is the angular rate at the start of target pointing.

The cost function used for generating an optimized profile for the maneuver segment in the second step is given by

(37) $$Cost\;function\;for\;optimization={\left(Quaternion\;error\right)}_{target\;pointing\;start\;time}+{\left(Rate\;error\right)}_{target\;pointing\;start\;time}.$$

**Figure 5:**
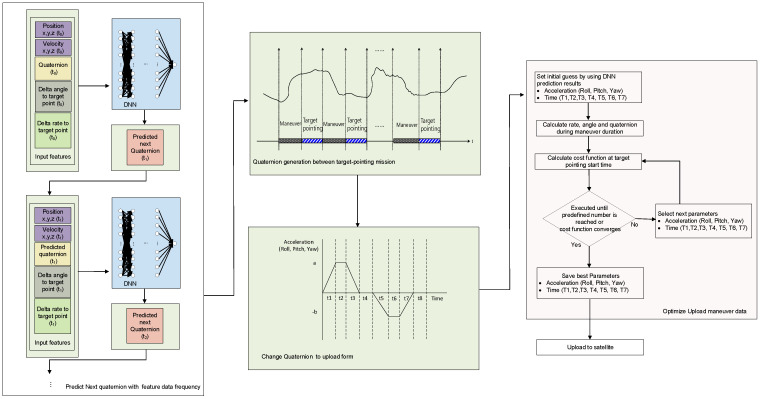
Maneuver reference profile generation for LEO satellites.

When applying the generated reference maneuver profile to an actual satellite’s attitude control logic, the additional errors that may arise during the restoration process must be considered. Thus, we included a margin in determining the convergence range of the cost function used for optimization, taking into account the potential additional errors. A quaternion error of 10^−4^ and a rate error of 10^−3^°/s were used for optimization. The process for generating the maneuver reference profile for LEO satellites is shown in Figure 5.

## 4. DNN Training Results

In this section, we summarize the quaternion prediction results of the data-based learning model generated in the first step. To verify the accuracy of the data-based learning model predictions, pointing maneuver profiles in the range of 2–68° were used as a reference for validation.

**Figure 6:**
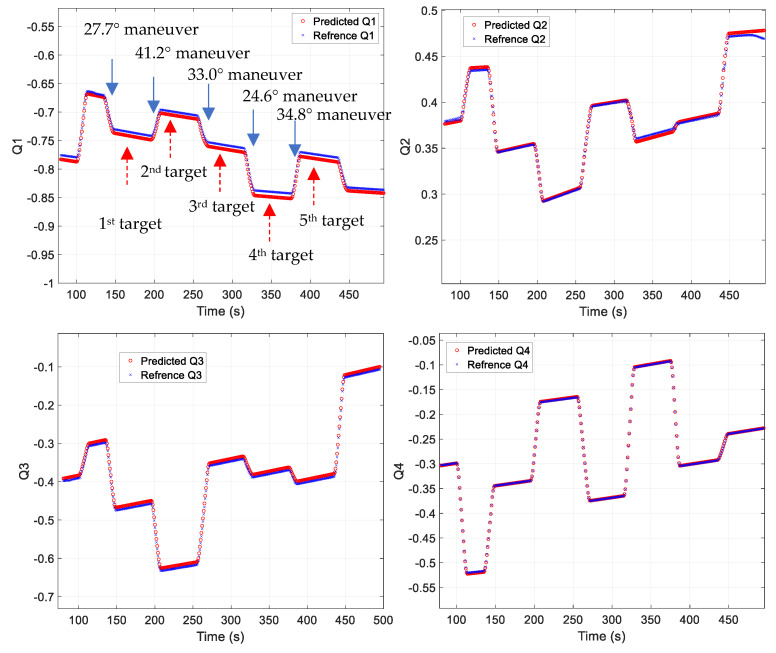
Quaternion prediction results.

Figure 6 shows the predicted quaternion results for a mission that performed continuous maneuvers at 27.7°, 41.2°, 33.0°, 24.6°, and 34.8°. As shown in the figure, similar predictions are possible for different target-pointing maneuvers. However, significant prediction errors occurred for target-pointing periods or particular attitude changes, such as a final sun-pointing maneuver. This study aimed to minimize the errors between target-pointing maneuvers; thus, the changes in the target pointings and attitudes were excluded from the training process, resulting in the observed performance. The data from the mission duration and certain attitude changes exhibited characteristics of the time-series data that were difficult to generalize. Therefore, including this data in the training step would result in performance degradation between pointing maneuvers. 

Because the maneuvers between target pointings were in the form of time-series data, it was difficult to judge the performance of the entire sequence based on the accuracy at a single point. In addition, when creating a maneuver profile, the attitude control performance was affected by the angular rate error at the final point of the maneuver. Furthermore, the conversion to the angular rate and time uploaded to the satellite was meaningful for the entire duration of the maneuver. Therefore, the root mean square (RMS) value of the maneuver duration was used to confirm the prediction performance.

**Figure 7:**
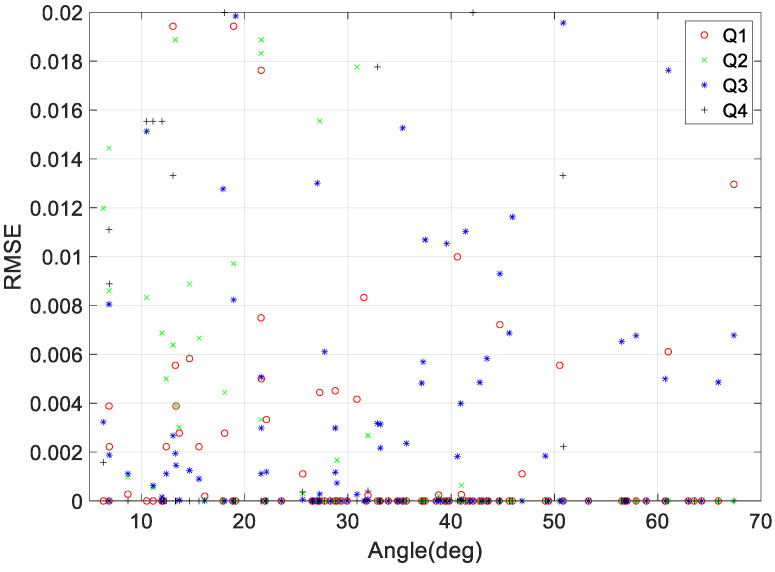
Quaternion prediction RMSE according to target pointing tilt angle.

As mentioned in Section 3, we used a dataset to validate the performance of the quaternion prediction model used to represent the maneuver duration based on various maneuvering angle conditions and differences in the angular rate at the initial and final points of the maneuver. Figure 7 shows the RMS error (RMSE) of the quaternion prediction model for various angle maneuvers, including roll, pitch, and yaw. As seen in the figure, there are minimal differences in performance depending on the maneuvering angle, and the prediction is accurate to an error of less than 0.02. 

## 5. Case Studies

In this section, we examine the generated LEO satellite maneuver profiles that conform to various initial and final boundary conditions, minimize maneuver time, and satisfy the constraints described in this paper. As mentioned earlier, when generating maneuver profiles solely using a DNN, the quaternion is generated within the error range. Therefore, the satellite attitude and angular rate estimated at the end of the maneuver can be affected not only by the attitude and angular rate error at the end of the maneuver but also by the error throughout the maneuver duration, which can lead to more significant estimation errors. Additionally, performance degradation may occur for certain initial and final attitudes, angular velocities, and angular accelerations. Therefore, we analyzed the results of the second-stage optimization correction using the predicted value from the DNN that was constructed using data-based learning as an initial guess. 

**Figure 8:**
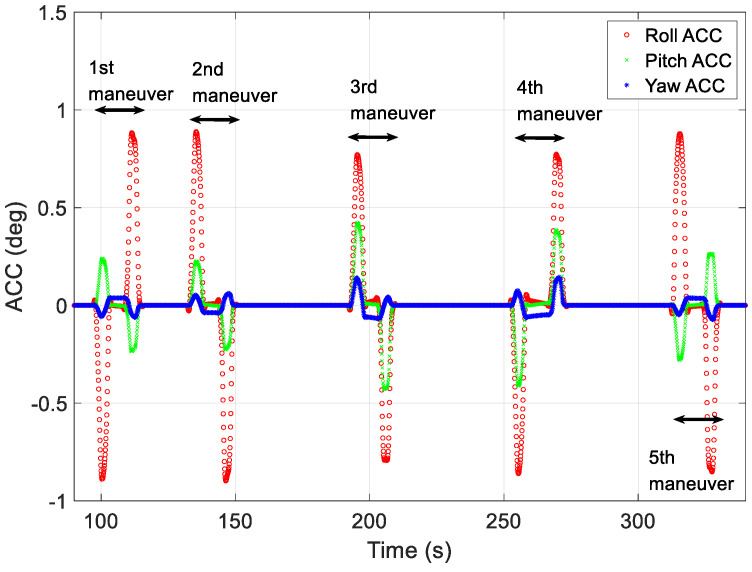
Angular acceleration profiles generated using proposed technique.

**Figure 9:**
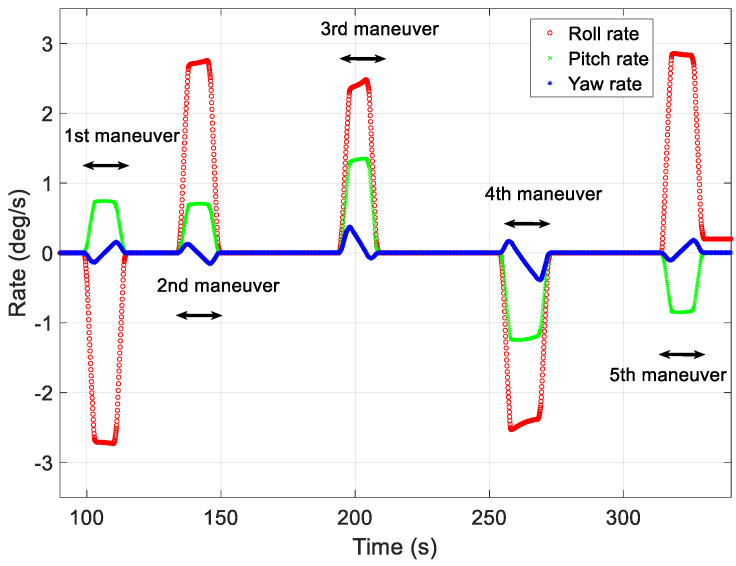
Angular rate profiles reproduced using proposed technique.

**Figure 10:**
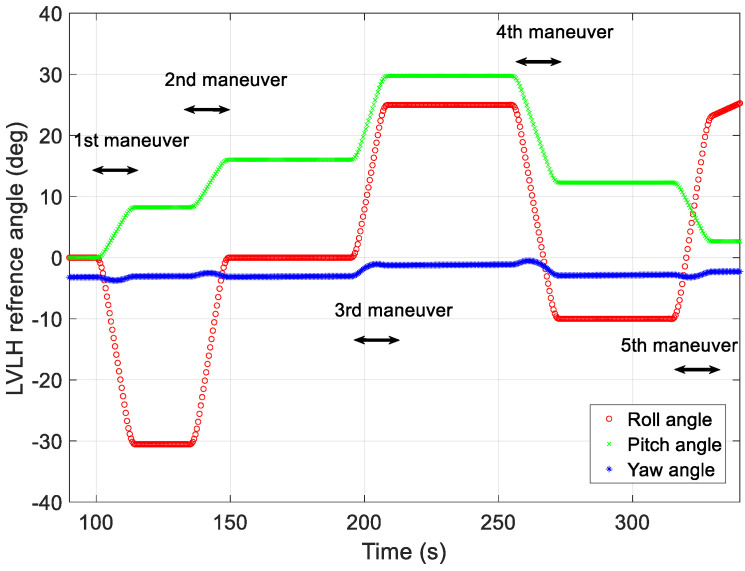
Angle profiles reproduced using proposed technique.

Figure 8, Figure 9 and Figure 10 show the maneuver profiles generated for the five missions. For the target-pointing segment, a maneuver range of 2–68° was assumed, and the angular velocities exhibited during the mission ranged from 0.01 to 0.25°/s. In the figures, the yaw maneuver for the mission duration was assumed to have the angle and angular rate of the pointing that was aimed at the same direction as the target, considering the Earth’s rotation and the roll and pitch exhibited various angles and angular velocities to point at different targets. 

The first target-pointing mission required a roll of −30.53°, a pitch of 8.24°, and a yaw of −3.04°. At the start of the first mission, the initial angular velocities for the roll, pitch, and yaw axes were 0.01°/s, 0.02°/s, and 0.05°/s, respectively. The second target-pointing mission required a roll of 30.54°, a pitch of 7.79°, and a yaw of −0.01°. At the start of the second mission, the initial angular velocities for the roll, pitch, and yaw axes were 0.03°/s, 0.01°/s, and 0.04°/s, respectively. The start of the third mission required a roll of 30.54°, a pitch of 7.79°, and a yaw of −0.01°. At the start of the third mission, the initial angular velocities for the roll, pitch, and yaw axes were 0.04°/s, 0.03°/s, and 0.06°/s, respectively. The fourth mission required a roll of −35.00°, a pitch of −17.45°, and a yaw of −1.67°. At the start of the fourth mission, the initial angular velocities for the roll, pitch, and yaw axes were 0.03°/s, 0.04°/s, and 0.08°/s, respectively. The final mission required a roll of 30.28°, a pitch of −9.61°, and a yaw of 0.63°. At the start of the fifth mission, the initial angular velocities for the roll, pitch, and yaw axes were 0.25°/s, 0.09°/s, and 0.05°/s, respectively. 

Figure 8 shows the angular acceleration profiles generated using the proposed technique for generating maneuver profiles for each of the five missions. To reduce the upload burden on the LEO satellite, angular accelerations and segment-specific time data were used to create profiles on the ground. In Figure 8, the red, green, and blue lines represent the angular acceleration on the roll-, pitch-, and yaw-axes in the local vertical local horizontal (LVLH) frame, respectively. The generated profiles were used to verify the angular rate at the start of the mission and the quaternion attitude error.

Figure 9 shows the angular rate profiles for the five target-pointing missions. The red, green, and blue lines represent the roll, pitch, and yaw rates in the LVLH frame, respectively. The angular velocities were generated by integrating the uploaded data, which included angular accelerations and time information. The error in each angular rate at the start of each target-pointing mission was less than 0.0001°/s, and significant discontinuities were not observed in the figure. 

Figure 10 shows the angle profile restored via the proposed method using the generated angular acceleration and time data. The red, green, and blue lines represent the roll, pitch, and yaw angles in the LVLH frame, respectively. The error of each angle was maintained at less than 0.007 at the beginning of each target pointing, and no significant discontinuities were observed in the figure. In addition, when the difference in the rate between the end of one mission and the beginning of the next was slight (i.e., there was an RMS difference of less than 0.1°/s) or significant (i.e., there was an RMS difference of more than 0.1°/s), both were generated within a similar error range.

**Figure 11:**
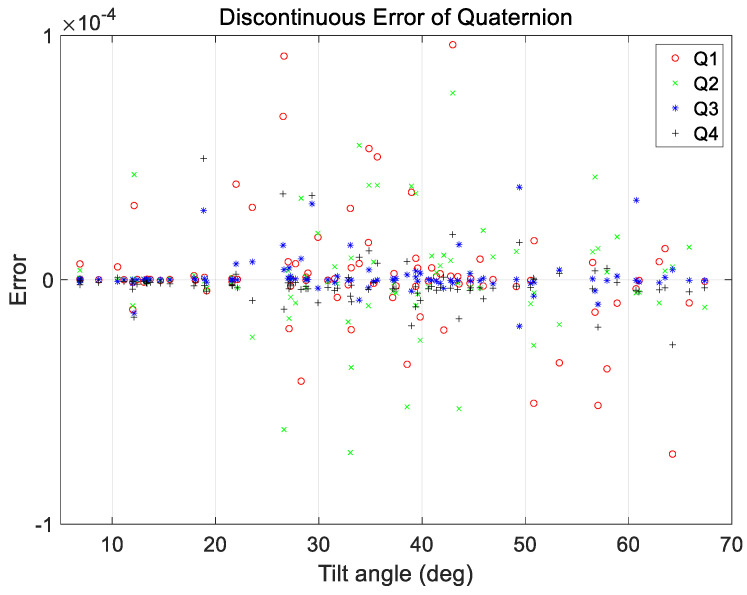
Discontinuous quaternion errors at initial position of target pointing generated by proposed technique as a function of maneuver angle.

**Figure 12:**
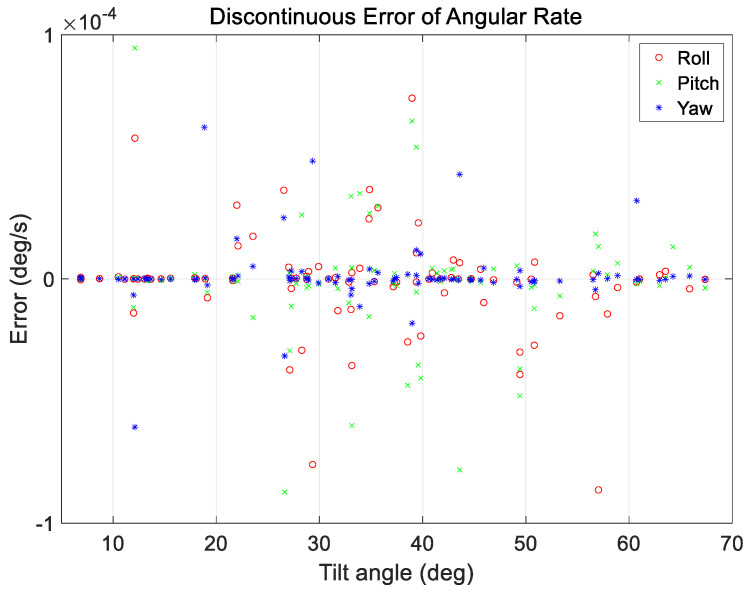
Discontinuous angular rate errors at initial position of target pointing generated by proposed technique as a function of maneuver angle.

**Figure 13:**
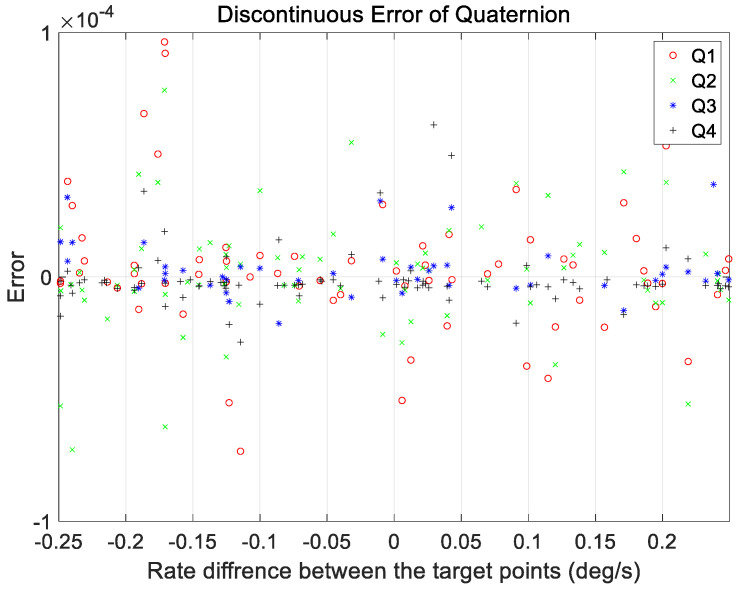
Discontinuous quaternion errors at initial position of target pointing generated by proposed technique as a function of satellite rate difference between target points.

**Figure 14:**
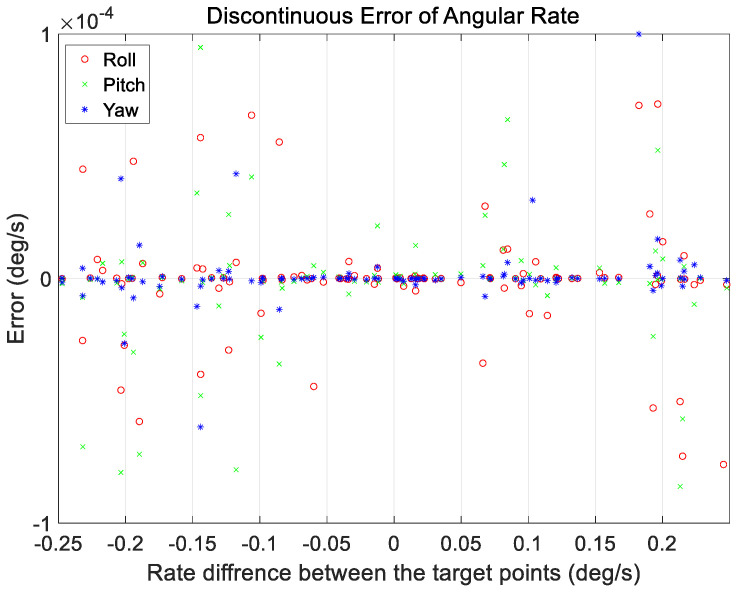
Discontinuous angular rate errors at initial position of target pointing generated by proposed technique as a function of satellite rate difference between target points.

An additional case analysis was performed to evaluate the performance under various maneuvering conditions. The test set used in the analysis considered a range of ground observation values with maneuver angles ranging from 2 to 68°. The angular rate range at the start and end of the maneuvers was set to a constant range of 0.01–0.25°/s for all maneuvers. As mentioned in Section 3, a total of 200 cases in the verification dataset were used for analysis, and the proposed technique was applied. The results are shown in Figure 11, Figure 12, Figure 13 and Figure 14.

Figure 11 and Figure 12 show the range of the quaternion and angular rate errors as a function of the maneuver angle, respectively. Figure 13 and Figure 14 show the quaternion and angular rate error values as a function of the difference in angular rate between the target pointings, respectively. As shown in the figures, the proposed technique generates the same performance for various maneuvering conditions without any significant degradation within the range of the quaternion and angular rate errors. 

**Figure 15:**
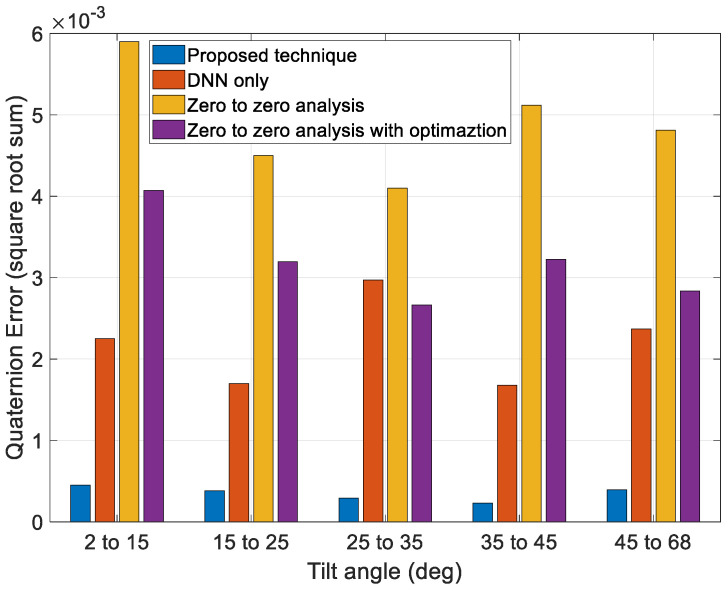
Discontinuous quaternion error at initial position of target pointing as a function of tilt angle.

**Figure 16:**
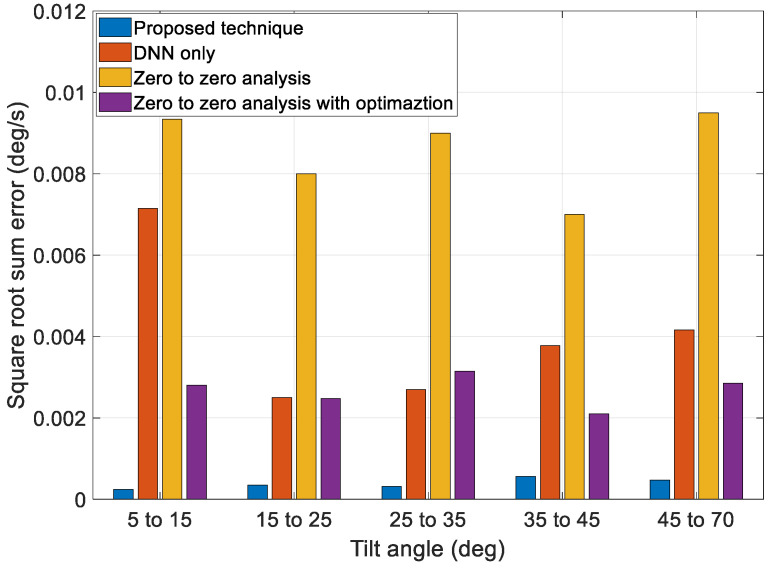
Discontinuous angle error at initial position of target pointing as a function of tilt angle.

Next, to validate the performance of the proposed technique, we compared the results of a zero-to-zero analysis, the optimization technique using a zero-to-zero analysis value as the initial guess, the profiles generated solely by the DNN, and the profiles generated by the proposed technique, as shown in Figure 15 and Figure 16. The zero-to-zero analysis is a conventional technique that can obtain a general solution using a simple calculation. The zero-to-zero analysis involves the generation of a motion profile by assuming that the angular rate of the target pointing moment is zero. This technique generates fewer errors when the angular rate of the target pointing duration is small, and it also requires fewer computations. However, it has limited coverage under various mission conditions. The optimization technique using a zero-to-zero analysis value as the initial guess can reduce errors more than the zero-to-zero analysis, but the performance is limited owing to the limit of the optimal starting point. 

Figure 15 and Figure 16 show that the overall error generated by the zero-to-zero analysis is more significant than that generated by the other techniques. Additionally, the performance of the profiles generated solely by the DNN and optimization using the zero-to-zero analysis to obtain an initial guess was better than that of the zero-to-zero analysis. However, some sections using the DNN technique solely exhibited worse performances. The optimization using the zero-to-zero analysis to obtain an initial guess had an error similar to that of only the DNN technique, but the performance was poor compared to that of the proposed technique. The proposed technique exhibited an overall smaller error range than the other techniques and had consistent performance under various maneuver conditions. 

## 6. Conclusions

For LEO satellites to perform observation missions and ground contact, it is essential to generate attitude profiles. Reference profiles for maneuvers between target pointings are directly related to the mission performance and accuracy of the antenna position to the ground contact. However, considerations, such as the initial and final boundary conditions (angles and angular velocities), constraint conditions (maximum jerk, angular acceleration, and angular rate), and throughput and upload capacity to be applied to the satellite must be taken into account. Therefore, this study used a data-based learning model and an optimization algorithm to develop a technique for generating reference profiles for the maneuvering phase of LEO satellites. The proposed technique demonstrated effective performance under various initial and final maneuvering conditions. In conclusion, it is expected that the proposed technique will optimize the LEO mission design performance and increase the satellite antenna pointing accuracy to the ground station.

## Figures and Tables

**Table 1:** Parameters of proposed technique for generating the reference attitude between target pointings.

	Parameter
Roll	Maximum acceleration, Minimum acceleration for roll axis
Pitch	Maximum acceleration, Minimum acceleration for pitch axis
Yaw	Maximum acceleration, Minimum acceleration for yaw axis
Time	Acceleration duration *t*_1_, *t*_2_, *t*_3_, *t*_4_, *t*_5_, *t*_6_, *t*_7_

**Table 2:** Input and output features for maneuver duration modeling of LEO satellites.

	Parameter	Number of Features
Input features	Current point quaternion	4
	Satellite position vector	3
	Satellite velocity vector	3
	Relative angle difference to target point	3
	Relative angular rate difference to target point	3
Output features	Next point quaternion	4

## Data Availability

The data is unavailable due to privacy.
